# Genome-Wide Identification of the LAC Gene Family and Its Expression Analysis Under Stress in *Brassica napus*

**DOI:** 10.3390/molecules24101985

**Published:** 2019-05-23

**Authors:** Xiaoke Ping, Tengyue Wang, Na Lin, Feifei Di, Yangyang Li, Hongju Jian, Hao Wang, Kun Lu, Jiana Li, Xinfu Xu, Liezhao Liu

**Affiliations:** 1 College of Agronomy and Biotechnology, Chongqing Engineering Research Center for Rapeseed, Academy of Agricultural Sciences, State Cultivation Base of Crop Stress Biology for Southern Mountainous Land, Southwest University, Chongqing 400715, China; xiaokeping1995@163.com (X.P.); tengyue1992@126.com (T.W.); linna123@yeah.net (N.L.); sddifeifei@163.com (F.D.); liyangyangswu@163.com (Y.L.); jianhongju1989@126.com (H.J.); drlukun@swu.edu.cn (K.L.); ljn1950@swu.edu.cn (J.L.); xinfuxu@126.com (X.X.); 2 Hybrid Rapeseed Research Center of Shanxi Province, Shanxi Rapeseed Branch of National Centre for Oil Crops Genetic Improvement, Yangling 712100, China; wangzy846@sohu.com

**Keywords:** laccase, *Brassica napus*, lignification, stress

## Abstract

Lignin is an important biological polymer in plants that is necessary for plant secondary cell wall ontogenesis. The laccase (*LAC*) gene family catalyzes lignification and has been suggested to play a vital role in the plant kingdom. In this study, we identified 45 *LAC* genes from the *Brassica napus* genome (*BnLACs*), 25 *LAC* genes from the *Brassica rapa* genome (*BrLACs*) and 8 *LAC* genes from the *Brassica oleracea* genome (*BoLACs*). These *LAC* genes could be divided into five groups in a cladogram and members in same group had similar structures and conserved motifs. All *BnLACs* contained hormone- and stress- related elements determined by cis-element analysis. The expression of *BnLACs* was relatively higher in the root, seed coat and stem than in other tissues. Furthermore, *BnLAC4* and its predicted downstream genes showed earlier expression in the silique pericarps of short silique lines than long silique lines. Three miRNAs (miR397a, miR397b and miR6034) target 11 *BnLACs* were also predicted. The expression changes of *BnLACs* under series of stresses were further investigated by RNA sequencing (RNA-seq) and quantitative real-time polymerase chain reaction (qRT-PCR). The study will give a deeper understanding of the *LAC* gene family evolution and functions in *B. napus.*

## 1. Introduction

*B. napus* originated from either the Mediterranean or Northern Europe and was formed by chromosome doubling after an interspecific natural cross between *B. rapa* (AA, 2n = 20) and *B. oleracea* (CC, 2n = 18) [1]. Rapeseed oil was once considered as a bad food choice because the seeds contain erucic acid and cholesterol, but with breeding selection and industrial improvement, *B. napus* has nowadays become the third largest source of vegetable oil. Unfortunately, *B. napus* is susceptible to various biotic and abiotic stresses, such as drought, heat, low temperature and fungi infection.

Lignin widely existed and composed of three monomers: coniferyl (G), sinapyl (S), and *p*-coumaryl (H) alcohols. Lignin in plants is involved in the formation of cell walls and together with cellulose increases cellular hardness. Studies have proved that lignin is related to drought stress [2] and a high content can improve the resistance to lodging and *Sclerotinia sclerotiorum* (*S. sclerotiorum*) [3,4]. Laccases are widely distributed with obvious functional differences in plants and fungi [5,6]. It can degrade lignin in *Pleurotus ostreatus* [7] and is expressed in lignifying cells in many plant species [8,9]. LACs are also named multicopper enzymes and supposed to catalyze lignin formation by polymerizing monolignols in plants [10].

To date, *LACs* have been characterized in many species. Lacquer tree contains LAC in the resin ducts and secreted resin [11]. In cotton, an ex-planta phytoremediation system was built based on the overexpression of *LACs* [12]. Forty-four, 46 and 84 *LACs* were identified from *Gossypium arboretum (G. arboretum), Gossypium raimondii (G. raimondii)* and *Gossypium hirsutum (G. hirsutum)*, respectively [13]. A total of 27 laccase candidates (*SbLAC1-SbLAC27*) were identified in *Sorghum bicolor* [14]. *LACs* are continually being detected in other species. An acidic *LAC* gene was found through cDNA cloning in sycamore maple and tobacco [15,16]. Five different LAC-encoding cDNA sequences were identified from ryegrass, with four from the stem and one from the meristematic tissue [17]. *Acer pseudoplatanus* has been found to produce and excrete LAC under cell culture [18,19]. In *Populus euramericana*, five distinct *LACs* were found in xylem tissue [20].

Many studies have indicated the relationship between LAC and lignification. In loblolly pine, LAC was purified from different xylem and shown to coincide with lignin formation in time and place. In *A. thaliana*, gene structure and molecular analysis of the laccase-like multicopper oxidase *(LMCO)* gene family noted that LAC genes *(AtLACs)*, *AtLAC4, AtLAC7, AtLAC8* and *AtLAC15* were mainly expressed in the seed coat, root, pollen grains and cell walls, respectively, and all these tissues present high lignification [21,22]. In maize (*Zea mays*), the *ZmLAC2, ZmLAC3, ZmLAC4*, and *ZmLAC5* coincided with the tissues undergoing lignification [23]. Northern blot analysis indicated that five *LACs* (*LAC1, LAC2, LAC3, LAC90* and *LAC110*) in poplar were highly expressed in stems, although their sequences vary greatly [20]. *SofLAC* was reported as a new *LAC* gene and proven to participate in lignification in sugarcane [24]. In *B. napus* and *Brachypodium distachyon* LAC has been shown to affect the accumulation of lignin [25,26]. In addition to oxidative lignin polymerization, LAC can also protect plants from biotic stresses and abiotic stresses such as the toxic phytoalexins and tannins in the host environment. In another study about maize, *ZmLAC3* was induced by wound, whereas *ZmLAC2* and *ZmLAC5* were repressed and *ZmLAC4* gene expression was unaffected [23]. The *OsChI1* gene encodes a putative *LAC* precursor protein in rice (*Oryza sativa*), overexpression of *OsChI1* in *A. thaliana* improved plants drought and salt tolerance [27]. Compared with the numerous reports about *LAC* gene in other species, few reports are available for *B. napus* especially at the genome level and the influence of stress on *BnLACs* [28,29].

In our study, we identified *LAC* gene family in *B. napus* and characterized them by gene structure, motif and cis-element analysis. The expression patterns of all *BnLACs* were identified by RNA -seq and some of them were analyzed under different stresses by RNA-seq or qRT-PCR. The research not only uncovers the evolutionary relationship of *LAC* gene family but also provides information about LAC respond to biotic and abiotic stresses.

## 2. Results

### 2.1. Characterization of the 45 BnLACs

Basic Local Alignment Search Tool Protein (BLASTp) was performed and confirmed 45 *BnLACs* in the *B. napus* genome by using 17 *AtLACs* protein sequences as queries (Table 1). Except for *AtLAC2, AtLAC8* and *AtLAC16,* the remaining *AtLACs* had more than one homologous gene in the *B. napu* genome. *AtLAC3, AtLAC4, AtLAC5, AtLAC11, AtLAC12* and *AtLAC17* had four homologs genes were the most. The genomic sequences lengths of *BnLACs* had a wide range from 1937 (*BnLAC13-1*) to 7114 bp (*BnLAC5-4*). The average MW is 63.02 kDa. The pI values of these proteins varied from 6.10 (*BnLAC9-1*) to 9.74 (*BnLAC14-2*). Subcellular localization predicted results showed all the 45 proteins are located in secretory except for *BnLAC11-4*, which is predicted in mitochondrion. Thirty-nine *BnLACs* are accurately, unevenly mapped on the 12 *B. napus* chromosomes and no tandem duplication. The remaining six *BnLACs* are located on the unmapped scaffolds in the Ann_random and Cnn_ random genome.

**Figure 1:**
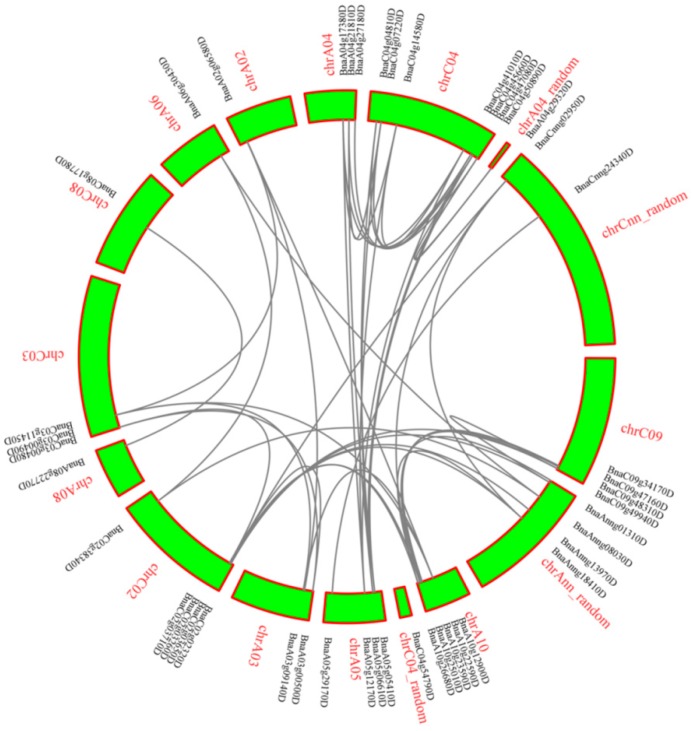
Interchromosomal relationships of *BnLACs* and gray lines indicate duplicated *LAC* gene pairs. The chromosome number and gene ID are indicated with red and black, respectively. The figure was generated by MCScanX with the default parameters [30].

**Figure 2:**
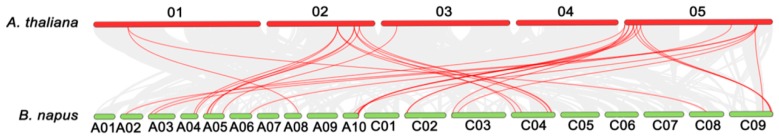
Synteny analysis of *LACs* between *A. thaliana* and *B. napus*. Gray lines in the background indicate all the collinear blocks, red lines highlight the syntenic *LAC* gene pairs. The figure was constructed by TBtools 0.66444553 (https://github.com/CJ-Chen/TBtools).

Chromosome C04 has the most *LACs* (eight) and A06, C08 only have one *LAC* gene (Figure 1). To further infer the phylogenetic mechanisms of *BnLACs,* a comparative syntenic map of *B. napus* associated with *A. thaliana* was constructed. Thirteen *BnLACs* show syntenic relationship with those in *A. thaliana* and focus on chromosome 02 and chromosome 05 (Figure 2).

### 2.2. Phylogenetic Analysis of LACs in A. thaliana, B. napus, B. rapa and B. oleracea

**Figure 3:**
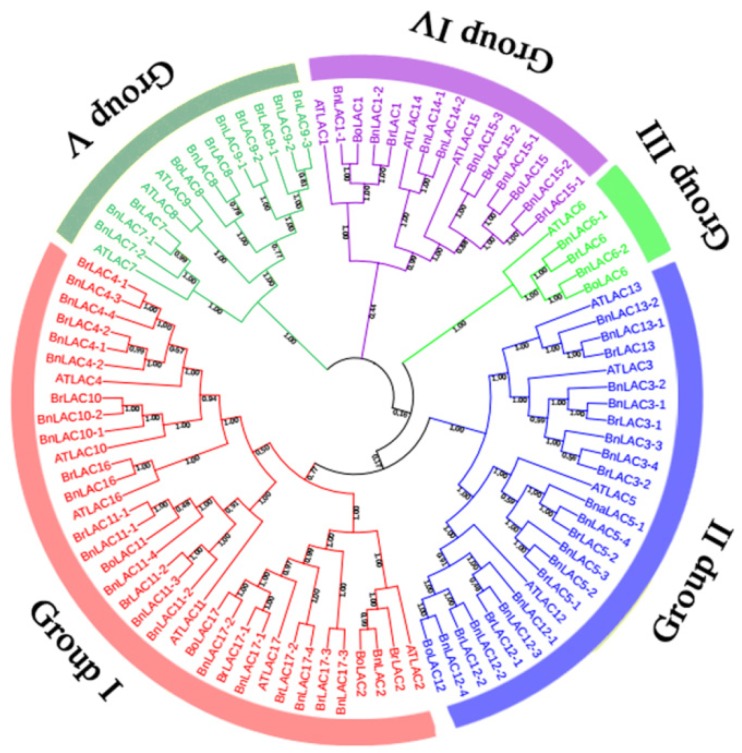
The cladogram of *LAC* proteins from *A. thaliana* (17), *B. napus* (45), *B. rapa* (25) and *B. oleracea* (8). The cladogram was constructed by the MEGA 7.0 software [31] using the neighbour-joining option with 1000 bootstrap replicates and pairwise deletion. Distinct colour segment represents different groups, bootstrap value are shown near nodes.

To study the evolutionary relationship of *LACs* in *A. thaliana, B. napus, B. rapa* and *B. oleracea*, a cladogram containing 45 *BnLACs*, 25 *BrLACs*, eight *BoLACs* and 17 *AtLACs* was constructed and divided into five groups with well-supported bootstrap values (Figure 3). Groups I, II, III, IV and V had 35, 26, 5, 15 and 14 members, respectively. Forty-five *BnLACs* were unevenly divided into five groups, the most being in Group I which contained 16 *BnLACs* the least being in Group III that only contained one. According to the bootstrap value in the tree, genes in same group were closely related but in different groups were far apart. Genes divided into the same group are thought to have similar functions and number of *LACs* in *B. oleracea* was far less than in *B. rapa* and *B. napus*, however, every group contained at least one *BoLAC*, which is essential to keep complete gene function of *LAC* in *B. oleracea*.

### 2.3. Gene Structure and Conservative Domain Analysis of BnLACs and AtLACs 

**Figure 4:**
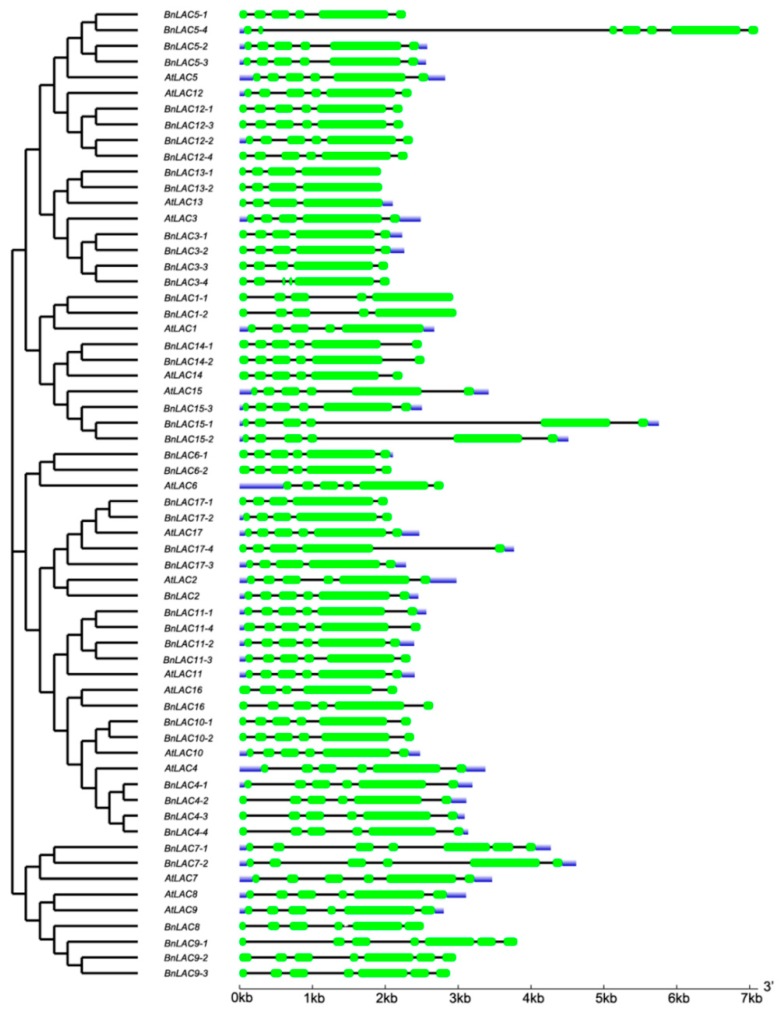
Exon-intron structures of *BnLACs* and *AtLACs* with a cladogram. The result generated by GSDS 2.0 (http://gsds.cbi.pku.edu.cn/). The green boxes, black lines and blue box indicate exons, introns, untranslated region, respectively.

**Figure 5:**
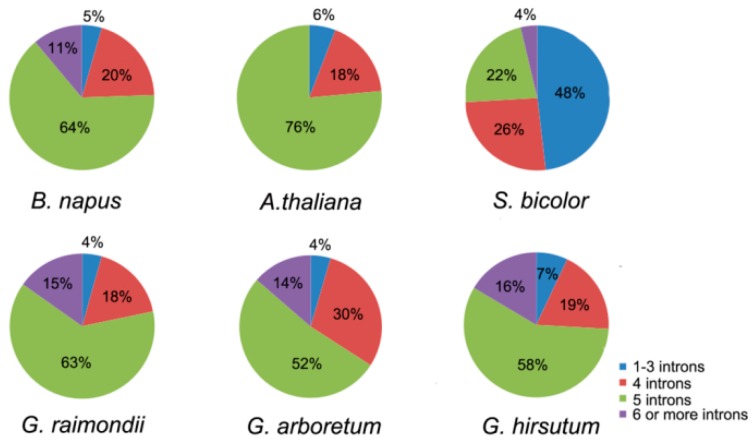
Number of introns in *A. thaliana, S. bicolor, G. arboretum, G. raimondii* and *G. hirsutum*. The figure was constructed by GraphPad Prism 6 (Graphpad Software Inc., La Jolla, CA, USA, www.graphpad.com).

As shown in the cladogram, members in the same group had highly similar gene structures, and the number of exons in the 45 *LACs* ranged from 4 to 7 (Figure 4). Compared with introns, exons were more stable in length. For example, *BnLAC5-4* had a separate longer intron but other members in the Group II didn’t contain one. *BnLACs* homologous with *AtLAC4, AtLAC7, AtLAC8* and *AtLAC9* showed diversity with other *BnLACs* because of one or two long introns existed. For exploring more characteristics about *LACs*, introns number of *LACs* from *A. thaliana, S. bicolor, G. arboretum, G. raimondii* and *G. hirsutum* were compared. In *A. thaliana*, there was no *LAC* gene had more six introns. Most *LACs* in *S. bicolor* have one to three introns less than other species with five introns (Figure 5).

**Figure 6:**
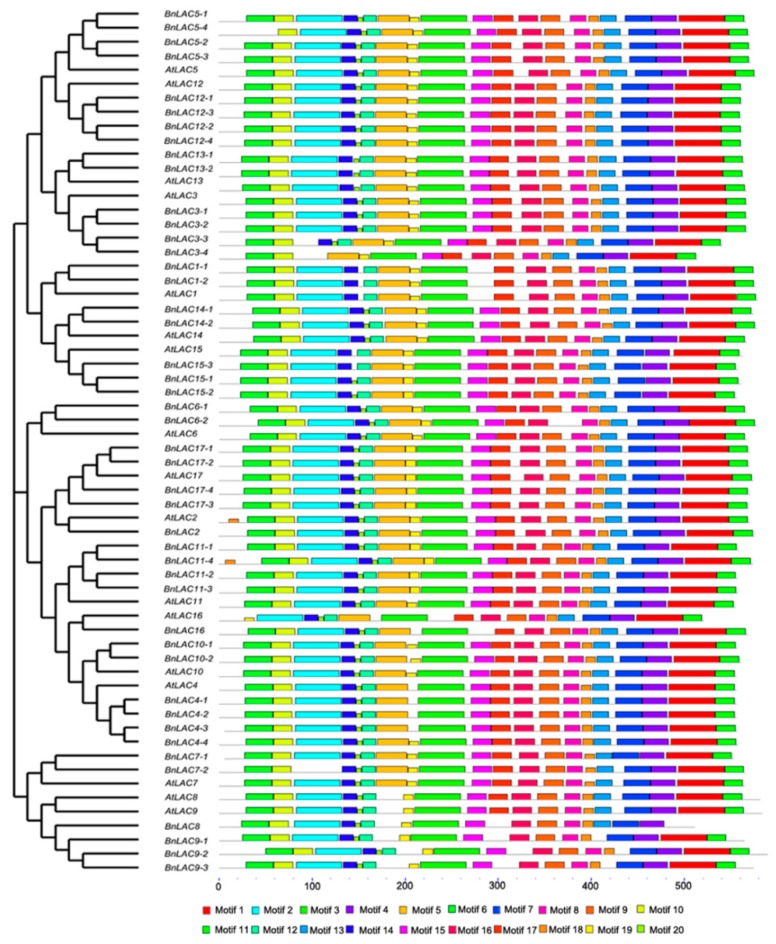
Conserved motif analysis of *BnLACs and AtLACs* proteins presented with a cladogram. Conserved motifs were generated by MEME 5.0.4 (http://meme-suite.org/tools/meme) and boxes of different colors represent motifs 1-20. Motif sequences are provided in Table S1.

Full-length protein sequences of 17 *AtLACs* and 45 *BnLACs* were analyzed to identify their conserved motifs and further understand their functions (Figure 6). The length of the 20 motifs ranged from 6 to 50 amino acids and motif sequences are provided in Table S1. *AtLAC4*, *BnLAC8* had the fewest motifs (eight), *BnLAC11-4* had the most motifs (twenty). In contrast to others *LACs, AtLAC2* and *BnLAC11-4* had an extra motif 18, *BnLAC3-3; BnLAC3-4* and *BnLAC7-2* lose the motif 2. *LACs* divided into the same groups also had different motifs, *BnLAC5-4* and *AtLAC16* lost the motif 6 compared with other genes in their corresponding group. *AtLAC8, AtLAC9* and their homologous gene in *B. napus* genome lacked the motif 5 when compared with others in Group V. Motif 1 and motif 3 are highly conserved and can be found in all the protein sequences of 62 *LACs*.

### 2.4. Diverse cis Regulatory Elements and miRNAs are Predicted

To investigate what condition could influence *BnLACs* expression, 1500 bp upstream of the initiation codons were analysed for cis-elements. Eleven kinds of elements including light responsive, six hormone-related, four stress-related elements were searched (Table S2). All the *BnLACs* are light and hormone responsive. Twenty-six *BnLACs* have a gibberellin element, 25 *BnLACs* have an ethylene element, which are the two most elements. Seven members contained a wound element and thirty *BnLACs* were influenced under drought stress. Fourteen members had low temperature elements and 34 members may have been influenced by heat stress.

Eleven *BnLACs* were predicted to have their expression regulated by miRNAs (Table S3, expectation number under 3.0 were selected). All the predicted *BnLACs* were regulated by miR397a and miR397b; *BnLAC4-1* and *BnLAC4-2* were also regulated by miR6034. As the expectation number showed, *BnLAC17-3* was the most likely targeted gene by miR397a and miR397b.

### 2.5. Expression Pattern Analysis of BnLACs

**Figure 7:**
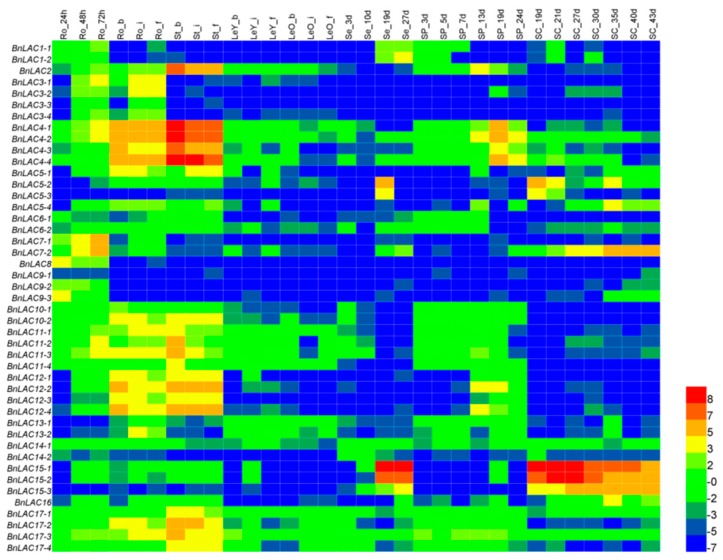
RNA-seq of *BnLACs* in *B. napus* 32 different tissues and stages. Ro, root; St, stem; LeY, young leaf; LeO, old leaf; Se, seed; SP, silique pericarp; SC, seed coat; s, seedling stage; b, bud stage; i, initial flowering stage; and, f, full-bloom stage. The 24, 48, and 72 h means the time that had passed after seed germination. The 3, 10, 27 d and other indicate the number of DAF. The bar on the lower right corner represents the Fragments Per Kilobase of Transcript Per Million Fragments Mapped (FPKM) values and different colors represent different expression levels. Heat map was generated by HemI 1.0 software (http://hemi.biocuckoo.org/faq.php).

To investigate the expression patterns of 45 *BnLACs*, a heatmap was built based on the RNA-seq (BioProject ID PRJNA358784) using 32 different tissues and stages of *B. napus* as samples (Table S4, Figure 7). Results suggested that *BnLACs* have different expression patterns across tissues and stages. Strong expression occurred in highly lignified tissues such as roots and stems. *BnLAC15-1* and *BnLAC15-2* had the highest expression in the seeds and seed coats. *BnLAC4-1, BnLAC4-2, BnLAC4-3* and *BnALC4-4* had high expression in silique pericarps. *BnLAC5-2, BnLAC5-3, BnLAC15-1, BnLAC15-2* and *BnLAC15-3* highly expressed in seed coats. No *BnLACs* highly expressed in leaves. *BnLAC9-1* and *BnLAC14-2* rarely expressed in any tissue and stage. These results showed *BnLACs* functioned differently and some members are redundant.

### 2.6. Responses of BnLACs upon Abiotic Stress

**Figure 8:**
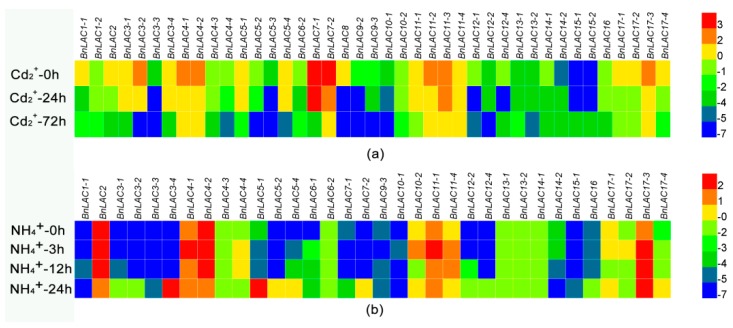
RNA-seq of *BnLACs* in *B. napus* tissues in response to Cd^2+^ stress (**a**), NH_4_^+^ stress (**b**). The 0, 3, 12 and other time points in (**a**,**b**) denotes the time after Cd^2+^, NH_4_^+^ stress respectively. The bar on the lower right corner represents the FPKM values and different colors represent different expression levels. Heat map was generated by HemI 1.0 software (http://hemi.biocuckoo.org/faq.php).

As many studies described lignification can response to stresses, RNA-seq and qRT-RCR were used to analyze the expression patterns of several *BnLACs* under different stresses. RNA-seq showed that under Cd^2+^ stress, *BnLAC14-2, BnLAC15-1* and *BnLAC15-2* were up-regulated, *BnLAC12-1* and *BnLAC12-4* were down-regulated at 24 hours after stress then up-regulated at 72 hours after stressed. *BnLAC6-1, BnLAC9-1* and *BnLAC15-3* had no change and other *BnLACs* were down-regulated at the analysed time points. Most *BnLACs* had low expression after 72 hours of Cd^2+^ stress and only 7 *BnLACs* (*BnLAC4-1, BnLAC4-2, BnLAC7-2, BnLAC11-2, BnLAC11-3, BnLAC11-4* and *BnLAC17-3*) still highly expressed (Table S5, Figure 8a). Under NH_4_^+^ toxicity, Expression levels of *BnLAC6-2, BnLAC13-1* and *BnLAC13-2* were low and not influenced by NH_4_^+^ toxicity. *BnLAC4-1, BnLAC10-2, BnLAC11-1* and *BnLAC17-2* were up-regulated at 3 or 12 hours after stress and changed to normal level at 48 hours. Some members responsed NH_4_^+^ toxicity until 48 hours after stressed, *BnLAC2, BnLAC4-2* and *BnLAC14-2* were down-regulated and members such as *BnLAC3-2, BnLAC3-3, BnLAC3-4, BnLAC5-2, BnLAC7-2, BnLAC12-2, BnLAC12-4* and *BnLAC15-1* show significant upregulation (Table S5, Figure 8b). Under drought and wound stress, expression levels of 14 *BnLACs* in stems and leaves of three lines (ZS11, 7191 and D2) were analyzed by qRT-RCR.

**Figure 9:**
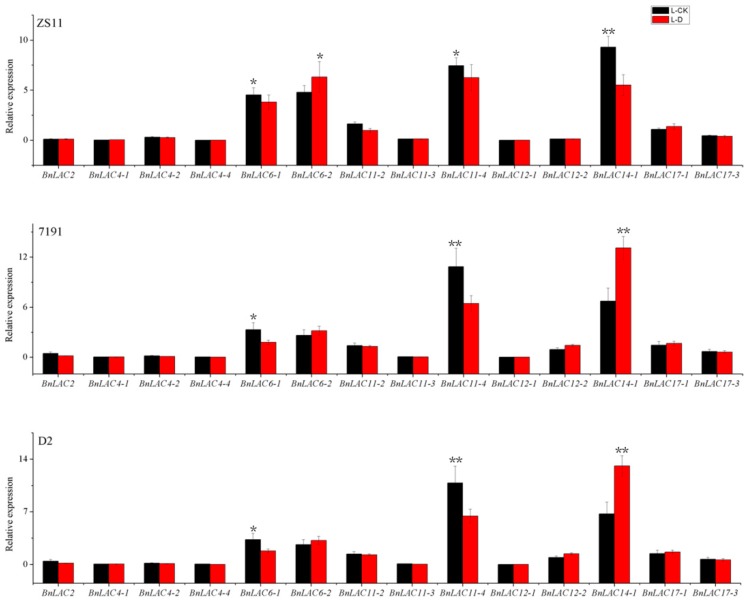
Quantitative RT-PCR of 14 *BnLACs* in *B. napus* leaves in response to drought stress. Expression of *Actin* and *UBC21* were used to normalize the expression level of each *LAC* gene. Bars represent means ± SEM of three biological replicates. Bars marked with asterisks indicate significant differences (Student’s t-test) to corresponding control samples for the same time point, *P < 0.05, **P < 0.01. The results were presented by OriginPro 8 (OriginLab Corporation, Northampton, MA, USA). L-CK and L-D means no drought treated and drought treated, respectively.

High expression concentrated at stems, which were consistent with the RNA-seq analysis (Table S6). In leaves of three lines under drought stress, the 14 *BnLACs* had similar expression pattern (Figure 9). *BnLAC2, BnLAC4-1, BnLAC4-2, BnLAC4-4, BnLAC11-3*, *BnLAC12-1, BnLAC12-2* and *BnLAC17-3* have stable expression and close to zero. *BnLAC6-1, BnLAC6-2, BnLAC11-2, BnLAC11-4, BnLAC14-1* and *BnLAC17-1* have relatively high expression. *BnLAC6-1, BnLAC11-2* and *BnLAC11-4* were down-regulated, *BnLAC6-2* and *BnLAC17-1* were slightly up-regulated under drought stress.

**Figure 10:**
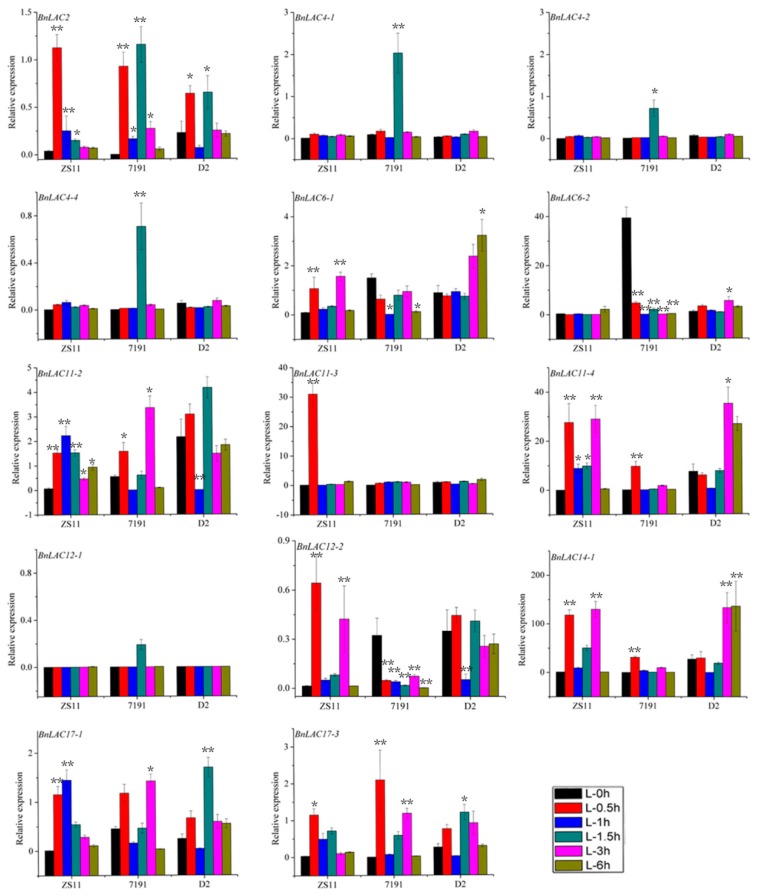
Quantitative RT-PCR of 14 *BnLACs* in *B. napus* leaves in response to wounding stress. Expression of *Actin* and *UBC21* were used to normalize the expression level of each *LAC* gene at each time point. Bars represent means ± SEM of three biological replicates. Bars marked with asterisks indicate significant differences (Student’s t-test) to corresponding control samples for the same time point, *P < 0.05, **P < 0.01. The results were presented by OriginPro 8. Different color means different time after wound shown as bar at lower right corner.

In wounded leaves of line ZS11, the expression of *BnLAC2, BnLAC11-2, BnLAC11-3*, *BnLAC17-1* and *BnLAC17-3* rose firstly then returned to normal levels. *BnLAC6-1, BnLAC11-4, BnLAC12-2* and *BnLAC14-1* had similar expression patterns and showed high expression at 0.5 and 3 hours after wounding. *BnLAC4-1, BnLAC4-2, BnLAC4-4, BnLAC6-2* and *BnLAC12-1* showed no expression in any samples. In wounded leaves of line 7191, *BnLAC4-1, BnLAC4-2, BnLAC4-4, BnLAC11-4* and *BnLAC12-1* were up-regulated followed by down-regulation and showed the highest expression at different time points. *BnLAC4-1, BnLAC4-2, BnLAC4-4* and *BnLAC12-1* showed the highest expression at 1.5 hours after wounding and *BnLAC11-4* showed the highest expression at 0.5 hours after wounding. *BnLAC6-2, BnLAC12-2* and *BnLAC14-1* were down-regulated for all the analyzed time points and *BnLAC11-3* had no change after wounding. In wounded leaves of line D2, *BnLAC2, BnLAC6-2 BnLAC11-2, BnLAC12-2, BnLAC17-1* and *BnLAC17-3* had high expression at two time points, 0.5 and 1.5 hours after wounding, respectively. *BnLAC4-1, BnLAC4-2, BnLAC4-4, BnLAC11-3* and *BnLAC12-1* nearly have no expression in the control sample and kept stable under wounding stress. Expression levels of *BnLAC6-1, BnLAC11-4,* and *BnLAC14-1* responded to wounds slowly and showed high expression at 3 and 6 hours (Figure 10).

**Figure 11:**
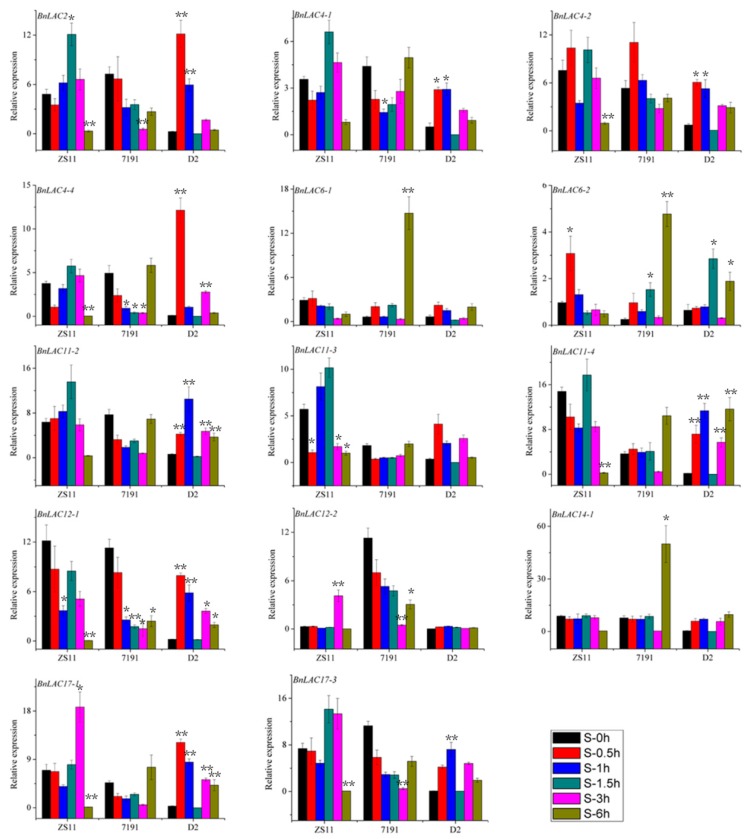
Quantitative RT-PCR of 14 *BnLACs* in *B. napus* stems in response to wounding stress. Expression of *Actin* and *UBC21* were used to normalize the expression level of each *LAC* gene at each time point. Bars represent means ± SEM of three biological replicates. Bars marked with asterisks indicate significant differences (Student’s t-test) to corresponding control samples for the same time point, *P < 0.05, **P < 0.01. The results were presented by OriginPro 8. Different color means different time after wound shown as bar at lower right corner.

In wounded stems of line ZS11, *BnLAC2, BnLAC4-1, BnLAC4-4, BnLAC11-3, BnLAC11-4* and *BnLAC12-1* had the highest expression at 1.5 hours after wounding and changed to zero at 6 hours. *BnLAC6-2, BnLAC11-2, BnLAC12-2, BnLAC17-1* and *BnLAC17-3* were up-regulated followed by down-regulation. *BnLAC6-1* and *BnLAC14-1* were down-regulated all the time after wounding. In wounded stems of 7191 line, *BnLAC4-1, BnLAC4-4, BnLAC11-2* and *BnLAC11-3* were down-regulated followed by up-regulation and showed high expression at 6 hours after wounding. *BnLAC12-1, BnLAC12-2* and *BnLAC17-1* were down-regulated at all the analyzed time points. *BnLAC6-1, BnLAC6-2, BnLAC11-4* and *BnLAC14-1* showed similar expression patterns and had their highest expression at 6 hours after wounding. In wounded stems of D2 line, *BnLAC2* and *BnLAC4-4* had the highest expression at 0.5 hour after wounding. *BnLAC4-1, BnLAC4-2, BnLAC11-3, BnLAC12-1, BnLAC17-1* and *BnLAC17-3* showed high expression at 0.5 and 1 hours after wounding. Expression levels of *BnLAC6-1, BnLAC12-2* and *BnLAC14-1* almost no change and were close to zero for the analyzed time points. Both *BnLAC11-2* and *BnLAC11-4* had the highest expression at 1 hour and lowest expression at 1.5 hours after wounding (Figure 11).

### 2.7. BnLAC4 and its Predicted Downstream Genes are Differentially Expressed in the Silique Pericarp between Long and Short Silique Lines

**Figure 12:**
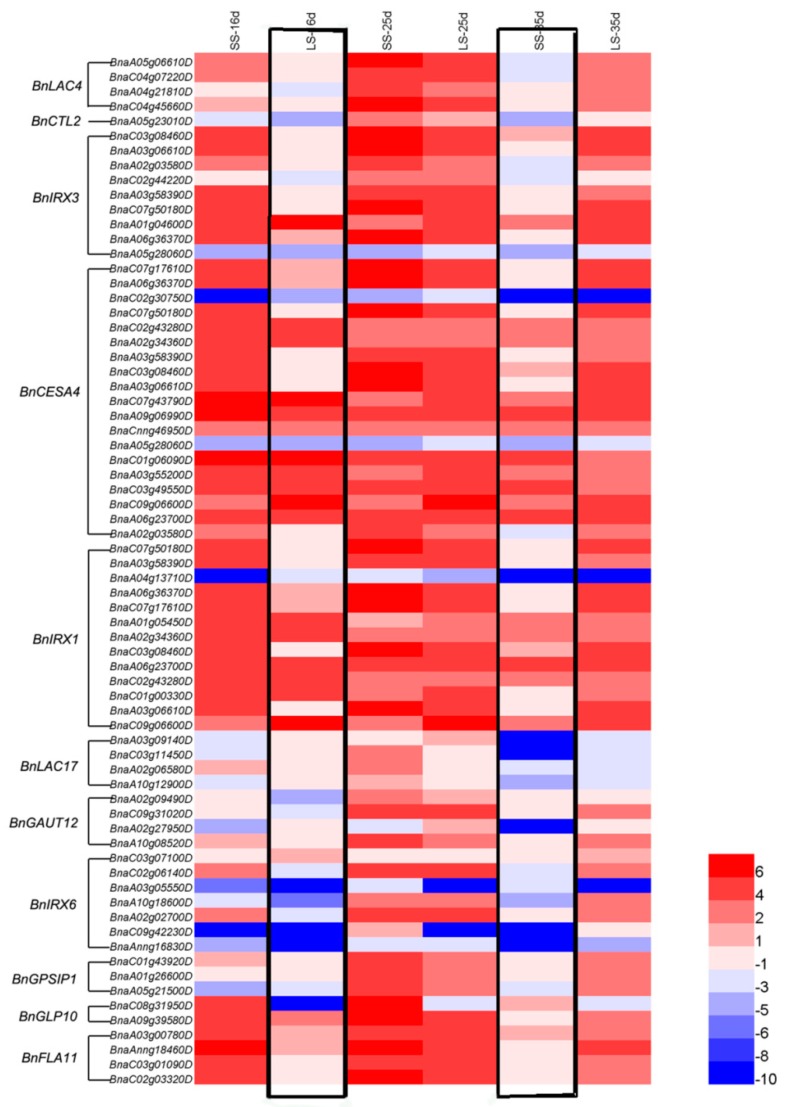
RNA-seq of BnLAC4 BnCTL2, BnIRX3, BnCESA4, BnIRX1, BnLAC17, BnGAUT12, BnIRX6, BnPGSIP1, BnGLP10 and BnFLA11 genes in six kinds of B. napus silique pericarps. LS, long silique lines; SS, short silique lines. The 16, 25, and 35 d means DAF. The bar on the lower right corner represents the FPKM values and different colors represent different expression levels. Heat map was generated by HemI 1.0 software (http://hemi.biocuckoo.org/faq.php). Lower expression between long and short silique lines at 16th and 35th DAF were indicated by black box.

In STRING platform, CTL2, IRX3, CESA4, IRX1, LAC17, GAUT12, IRX6, PGSIP1, GLP10 and FLA11 were predicted to interact with protein AtLAC4. A total 70 homologous genes were identified in the B. napus genome and expression levels of them in silique pericarps of long and short siliques lines were showed by a heatmap (Table S7, Figure 12). Most of the identified genes showed higher expression in silique pericarps of short silique lines on the 16th Days After Flower (DAF) and almost equal expression in two kinds of silique pericarps on the 25th DAF. On the contrary, many genes showed a higher expression in long silique lines on the 35th DAF. Those results showed BnLAC4 and its’ predicted downstream genes expressed earlier in silique pericarp of short siliques lines.

## 3. Discussion

*B. napus* is the third largest source of vegetable oil worldwide and plays an important role in national economies and food industries. The rapeseed yield decreases frequently because of lodging and other biotic and abiotic stresses. Many studies have shown that lignin aids in the resistance to fungi, stress and the LAC acts as an enzyme related to lignification in plants. Previous studies on the *LAC* gene family have been performed, but no related reports on the analysis of this family in *B. napus* exist until now. We identified and analyzed the LAC gene family with the aim of providing a reference at the genome level and deeper understanding of lignification.

### 3.1. Loss Events Occurred in the LAC gene family Along with the Evolution

Loss events occur frequently during evolution because of hybridization and chromosome doubling [32]. As a result of whole-genome triplication (WGT), the genes in *A. thaliana* should have three homologs in *B. rapa* and *B. oleracea.* In the study, only *AtLAC1* had three orthologous genes in *B. rapa* and no *AtLACs* corresponding to three orthologous genes were found in *B. oleracea*. Some *AtLACs* even have no orthologous genes in *B. rapa* and *B. oleracea* like *AtLAC14* in *B. rapa*, *AtLAC3* and *AtLAC4* in *B. oleracea*. Furthermore, *B. napus* formed by natural hybridization and polyploidization of *B. rapa* and *B. oleracea*, but no one *AtLAC* corresponding to six *BnLACs* was seen in this study and the most is four. Hence, the conclusion can be drawn that not only during the whole-genome triplication but also the formation of *B. napus*, gene loss events existed in *LAC* gene family universally.

Twenty four *BoLACs* were identified in *B. oleracea,* but only eight of them contained four essential conserved domains as described in the Materials and Methods section. Compared with *B. rapa* that contained 25 *BrLACs*, faster or broader gene loss happened in *B. oleracea*.

### 3.2. Regulation of BnLAC Genes

Cis-element analysis showed the expression of *BnLACs* was regulated at transcriptional level (Table S2). Research has shown *LAC* genes were also regulated at the post-tanscriptional level or through post-translational modifications [14]. G-box can be found in some *BnLACs* and related to light-response and salt tolerance in rice flag leaf by combinating with bZIP, bHLH, and NAC TFs [33,34,35]. The *AtLAC4* gene has been confirmed to be up-regulated after MYB58 binds to AC elements [36]. Our results show diverse cis-elements in promoters of *BnLACs*, 11 kinds of promoters were selected for analysis including light responsive element, six hormone-related elements and four stress-related elements (Table S2). These findings revealed that *BnLACs* are also regulated by a series of factors. Related studies have reported that Ptr-miR397a is a negative regulator of *LACs* in *Populus trichocarpa* [37]. Several *LACs* are targeted by miR408, miR397, and miR857 in *A. thaliana* when Cu is absent [38]. Ptr-miR397a and Os-miR397 are involved in negative regulation of *PtrLACs* and *OsLACs* [38,39]. Seven *SbLACs* have also been predicted to be sbi-miRNA targets [14]. In the study, 11 *BnLACs* were predicted to be regulated by miR397a, miR397b and miR6034 (Table S3). All the 11 predicted *BnLACs* were regulated by miR397a and miR397b, and *BnLAC4-1* and *BnLAC4-2* were also regulated by miR6034. Research about miR6034 is very few and the process it participates in is not clear. Results have proved *BnLAC17-3* was the most likely targeted gene by miR397a and miR397b in our study and in *A. thaliana* [40]. The findings of the study and combined with previous researches suggest *LAC* genes are truly regulated by miRNAs, and miRNA397 likely plays a very important role in the regulatory network.

### 3.3. Expression Patterns and Response to Stress

Abundant expression focuses on the roots, stems, and seed coats, whereas the expression in leaves, petals, pistils, and stamens are very low. The expression coincides with lignification in different parts of the plant. *BnLAC13-1* and *BnLAC13-2* showed different expression pattern though contain same cis-elements (Table S2, Figure 7). The reason would be a network containing other factors exists and regulates expression patterns of *BnLACs* such as miRNA and epigenetic modifications [41]. Some members, such as *BnLAC9-1* and *BnLAC14-1,* were never highly expressed in any tissues or stages. It might be that their function was not required in biological processes or was only induced. by certain environmental factors, similar genes can also be found in cotton and Sorghum bicolor [13,14].

Many studies have illustrated that *LACs* are influenced by different kinds of stress and our study also proved that. *OsChL1* as a putative laccase precursor, its expression was increased under drought stress and overexpressed the gene in *A. thaliana* can increase drought and salt tolerance [27]. In the study, six out of 14 *BnLACs* were influenced by drought stress. *BnLAC6-2, BnLAC11-2* and *BnLAC11-4* were up-regulated, *BnLAC6-1* and *BnLAC11-4* were down-regulated. *BnLAC14-1* was up-regulated in line 7191 and D2 but down-regulated in line ZS11. Metal ions would influence the expression of *LACs* and miRNAs directly or indirectly. In *Citrus*, the expression of *LAC7* was up-regulated by boron toxicity [42]. miR397 has been confirmed as a regulatory factor of *LACs* and its expression were influenced by Cd^2+^ [43]. In our study, most *BnLACs* were down-regulated after Cd^2+^ treatment and only four *BnLACs* showed upregulation. Another study in our lab has indicated NH_4_^+^ enrichment treatment would increase the lignin content in stem and root. Consistent with phenotype, the expression of most *BnLACs* were up-regulated at different time after NH_4_^+^ enrichment treatment by RNA-seq. According to the results of the promoter analysis, some members like *BnLAC5-1, BnLAC6-1, BnLAC6-2* contain cis element about wound and a study has been reported *LACs* were influenced by wound [23]. qRT-RCR results showed the expression of the selected 14 *BnLACs* in leaves and sterms changed intricately after wounded. We also found gene in different lines responded to stress differently, *BnLAC11-4* was up-regulated in lines 7191 and D2 but down-regulated in line ZS11. Further works are needed to find out the relation between wound healing and *LACs*.

### 3.4. BnLAC4 and its Downstream Genes May Participate in Silique Elongation in B. napus

Studies have reported that miR397 (both miR397a and b) regulate lignin content and yield traits in *Rice* and *Populus trichocarpa* via modulating *LACs* [39]. In *A. thaliana,* overexpression of miR397b-resistant *AtLAC4* results in an increased silique length and decreased lignin content [40]. In our research, homologous genes of *AtLAC4, AtCTL2, AtIRX3, AtCESA4, AtIRX1, AtLAC17, AtGAUT12, AtIRX6, AtPGSIP1, AtGLP10* and *AtFLA11* in *B. napus* show earlier expression in silique pericarp of short silique lines than long silique lines. Some of those genes predicted like *AtIRX1* and *AtIRX6* are related to secondary cell wall biosynthesis. Combined with the reports, it could be that the period of *BnLAC4* and its downstream genes expression may regulate silique length in *B. napus*.

## 4. Materials and Methods

### 4.1. Plant Materials and Stress Treatment

Inbred line Zhongshuang11 (ZS11), 7191, and D2 were sown in humus and grown to the four-leaf-stage. Half of the plants in each line were transplanted for drought stress and the remaining were left for the control. After 25 days without irrigation, the lines in the drought stress treatment showed a wilted phenotype and young leaves from control and stressed lines were frozen immediately in liquid nitrogen and stored at −80 °C.

Leaves and stems were wounded at the bolt stage. Leaves were wounded by a plastic comb-like brush, which was 8.5 cm long and had 42 spikes with a diameter of 1 mm that were equally arrange. Every leaf received three rows of wounds on each side of the midrib and parallel with it; the total number of punctures in each leaf was 252. Stems were wounded to a centimeter depth by scalpel blades. Samples were harvested around the cut at 0.5, 1, 1.5, 3, and 6 hours after wound [44]. The collected samples were frozen immediately in liquid nitrogen and stored at −80 °C for RNA isolation.

The seeds were grown at 22 °C with a light intensity of 200 mol/m^2^/s and a photoperiod of 16 hours for 7 days in hydroponic culture. Subsequently, the plants were collected after exposure to 1 mM Cd^2+^ (CdCl_2_) at 0, 24, and 72 h immediately frozen in liquid nitrogen for RNA sequencing.

Seeds of the *B. napus* line ZS11 were surface-sterilized with 1.2% sodium hypochlorite and germinated in a chamber room (16 hours light 15000Lx/8 hours dark at temperature 25 °C) with Hoagland solution. At four-leaf-stage, a portion of the seedlings were cultivated with modified Hoagland solution (0 mM NO_3_^−^, 10 mM NH_4_^+^, pH 6.0; other ions were not changed) for NH_4_^+^ toxicity treatment. After 3, 12 and 48 hours, the third and fourth true leaves were immediately frozen in liquid nitrogen and stored at −80 °C for RNA sequencing.

Long and short silique lines were selected from a recombinant inbred line (RIL) population constructed from a cross between GH06 (female parent) and P174 (male parent). Lines were planted in open field and grew under normal condition, silique pericarps of two kinds lines were collected respectively at 16th, 25th and 35th DAF and frozen in liquid nitrogen for RNA sequencing.

### 4.2. Characterisation of the LAC Gene Family

To date, 17 *LACs* have been reported in *A. thaliana* [35]. BLASTp was performed in the *B. napus*, *B. rapa* and *B. oleracea* genome using the *AtLACs* protein sequences as queries and sequences with E-value less than 1 × 10^-20^ were selected [45]. Some repeated sequences were manually deleted according to the E-value. All the remaining genes were checked by InterProScan (http://www.ebi.ac.uk/interpro) [46], and the sequences with four essential conserved domains of multicopper oxidase type 1 (IPR001117), multicopper oxidase type 2 (IPR011706), multicopper oxidase type 3 (IPR011707) and laccase (IPR017761) were deemed as candidate *LACs* [13]. Another BLASTp was performed in *A. thaliana* genome using candidate *BnLACs* protein sequences as queries and hold those genes that corresponded to *AtLACs*. *LACs* identified from *B. napus*, *B. rapa* and *B. oleracea* were named according to the orthologous sequence in *A. thaliana*. Information about putative sequences were searched in the date bases of BRAD (http://brassicadb.org/) and *B. napus* Genome Browser (http://www.genoscope.cns.fr/brassicanapus/). The chromosomal locations were shown by MapChart software [47], and the number of amino acids, isoelectric point (pI) and molecular weight (MW) of the protein sequences were searched using the ExPASy website (http://web.expasy.org/). The subcellular localization pattern of *LAC* genes were predicted using the web-based tool TargetP1.1 server (http://www.cbs.dtu.dk/services/TargetP/) [48]. Multiple Collinearity Scan toolkit (MCScanX) was adopted to analyze the gene duplication events with the default parameters [30]. The synteny relationship of the *LACs* in *B. napus* and *A. thaliana* were constructed using the Dual Systeny Plotter software (https://github.com/CJ-Chen/TBtools).

### 4.3. Evolutionary relationship of the LAC Genes Family in A. thaliana, B. napus, B. rapa, and B. oleracea

A cladogram containing the sequences identified from the four species was built using MEGA 7.0 software [31], with 1000 bootstrap replicates performed, and it was then modified by iTOL (http://itol.embl.de/) and Photoshop CS 5 to further visualize evolutionary relationship.

### 4.4. Gene Structure and Conserved Motif Analysis

The cDNA sequences, genomic sequences and full-length protein sequences of *AtLACs* and *BnLACs* were obtained from the *A. thaliana* genome (http://www.arabidopsis.org/) and *B. napus* Genome Browser (http://www.genoscope.cns.fr/brassicanapus/) respectively. Gene structures were analysed by Gene Structure Display Server (GSDS2.0, http://gsds.cbi.pku.edu.cn/) [49], conserved motifs were tested by and Multiple EM for Motif Elicitation version 5.0.4 (MEME, http://meme-suite.org/tools/meme) with a limit of 20 motifs and any number of repetitions deemed as motif sites [50].

### 4.5. Cis-Elements Analysis and Prediction of miRNA Target BnLACs

One thousand and five hundred base pairs (bp) upstream of the initiation codons (ATG) were searched in the *B. napus* Genome Browser and cis-elements were analysed using the PlantCARE database (http://bioinformatics.psb.ugent.be/webtools/plantcare/html/). The genome sequences of the 45 *BnLACs* were submitted to the psRNATarget Server (http://plantgrn.noble.org/psRNATarget/) with default parameters to predicte the miRNAs with a target site on *BnLACs*. MiRNAs from the *B. napus* genome were selected and expectation number under 3 were selected [51].

### 4.6. Expression Patterns Analysis of B. napus LAC Genes

The expression patterns of *BnLACs* were based on RNA-seq, using data from the BioProject ID PRJNA358784. The data included the expression in different tissues in different stages of the *B. napus* cultivar ZS11. The clean reads were aligned to the *B. napus* reference genome and these sequence data and corresponding gene annotation files were downloaded from the genome website (http://www.genoscope.cns.fr/ brassicanapus). The BWA and Bowtie softwares were used to map the reads to a reference genome and the reference genes, respectively [52]. The alignment results were visualized by IGV (Integrative Genomics Viewer) and genes expression levels were quantified on the basis of their FPKM values using Cufflinks with default parameters. For RNA-seq data about Cd^2+^ and NH_4_^+^ stresses, HISAT2 was used to map the reads to a reference genome and genes [53]. The alignment results were also visualized by IGV and the level of each gene expression was measured as FPKM by StringTie [54]. The heatmaps were built to represent the expression level of the *BnLACs* using HemI 1.0 software (http://hemi.biocuckoo.org/faq.php) [55].

### 4.7. RNA Extraction, Reverse Transcription and qRT-PCR

Total RNA was extracted using the EZ-10 DNAaway RNA Mini-prep Kit (Sangon Biotech, Shanghai, China). NanoDrop 2000 (Thermo Fisher Scientific, Worcester, MA, USA) and electrophoresis were used to measure concentrations and RNA integrity. Complementary DNA was obtained using the iScriptTM cDNA Synthesis Kit (Bio-Rad, Hercules, CA, USA) and diluted 15 times with distilled deionized water for qRT-PCR. The composition of qRT-PCR contained 2 µL of 15-fold diluted cDNA solution, 10 µL of SYBR® Green Supermix (Bio-Rad), 0.4 µL of 10 mM forward and reverse primers and 7.2 µL of distilled deionized water. Primers were designed on Primer Premier Software (version 5.0) (Table S8) [56] and qRT-PCR was performed on a CFX96 Real-time System (Bio-Rad) with the following conditions: 98 °C for 30 s, then 40 cycles of 98 °C for 15 s, 55 °C for 30 s, and an increase from 65–95 °C at increments of 0.5°C every 0.05 s. Three biological replicates and three technical replications were used for qRT-PCR. According to the 2^−ΔΔCt^ method using Actin7 and UBC21 as internal controls, the gene expression levels were determined and displayed by OriginPro 8 (OriginLab Corporation, Northampton, MA, USA).

### 4.8. Proteins Interaction with AtLAC4 and Identified their Homologous Genes in the B. napus genome

MiR397b regulated both lignin content and silique length via modulating *AtLAC4* has been identified [39]. To understand whether the similar interaction exit in *B. napus*, the protein sequences of *AtLAC4* was obtained from the *A. thaliana* genome (https://www.arabidopsis.org/) and used to predict the interacting proteins in STRING platform (https://string-db.org/?tdsourcetag=s_pctim_aiomsg). Homologous genes of predicted protein sequences were searched in the *B. napus* genome as the method of identifying *BnLACs*.

## 5. Conclusions

A total of 45 putative *BnLACs* were identified in the *B. napus* genome and unevenly mapped on the 12 *B. napus* chromosomes with no tandem duplication. *BnLACs* were divided into five groups in the cladogram and members in same group had similar structures and motifs. *BnLACs* had high expression in lignified tissues such as roots, stems and seed coats. After high concentration of NH_4_^+^ toxicity, most *BnLACs* were up-regulated and lignin more and faster deposited. Expression of many *BnLACs* were close to zero in leaves and uninfluenced by drought stress. Some *BnLACs* were down- regulated and individual gene showed different responses in different lines. Many members were intricately influenced by wounding stress, and significantly regulated members may take part in the healing process. By RNA-seq between long and short silique lines, we forecasted that earlier lignification may be a reason for the short siliques. The results in the study give a chance to further study the functions of *BnLACs* in lignification and the interaction with other biological processes.

## Figures and Tables

**Table 1:** Characterization of *BnLACs* identified in *B. napus* genome.

LAC	Gene ID	Predicted Subcellular Location	Chr	Position	Genomic Sequences Length (bp)	cDNA Length (bp)	Protein Sequences Length (aa)	MW (kDa)	pI
*BnLAC1-1*	*BnaC08g17780D*	Secretory	C08	21234694:21237623	2929	1737	578	65.20	9.09
*BnLAC1-2*	*BnaA08g22770D*	Secretory	A08	16433641:16436613	2972	1740	579	65.23	9.09
*BnLAC2*	*BnaC04g54790D*	Secretory	C04	2100118: 2102571	2453	1737	578	64.46	9.53
*BnLAC3-1*	*BnaA04g17380D*	Secretory	A04	14120708:14122939	2231	1713	570	64.07	9.48
*BnLAC3-2*	*BnaC04g41010D*	Secretory	C04	41756886:41759146	2260	1713	570	64.12	9.59
*BnLAC3-3*	*BnaA05g12170D*	Secretory	A05	6998148: 7000182	2034	1632	543	60.91	9.41
*BnLAC3-4*	*BnaC04g14580D*	Secretory	C04	11956954:11959010	2056	1551	516	57.83	9.63
*BnLAC4-1*	*BnaA05g06610D*	Secretory	A05	3604302: 3607497	3195	1677	558	61.54	9.36
*BnLAC4-2*	*BnaC04g07220D*	Secretory	C04	5406354: 5409465	3111	1677	558	61.54	9.36
*BnLAC4-3*	*BnaA04g21810D*	Secretory	A04	16546132:16549220	3088	1680	559	61.79	9.41
*BnLAC4-4*	*BnaC04g45660D*	Secretory	C04	45245585:45248722	3137	1683	560	61.88	9.41
*BnLAC5-1*	*BnaC04g47080D*	Secretory	C04	46166799:46169078	2279	1710	569	63.00	8.57
*BnLAC5-2*	*BnaA05g05410D*	Secretory	A05	2791689: 2794263	2574	1725	574	63.65	8.92
*BnLAC5-3*	*BnaC04g04810D*	Secretory	C04	3510057: 3512615	2558	1725	574	63.52	8.85
*BnLAC5-4*	*BnaA04g29320D*	Secretory	A04	1313319: 1320433	7114	1722	573	63.47	8.57
*BnLAC6-1*	*BnaA04g27180D*	Secretory	A04	19140832:19142936	2104	1710	569	63.62	8.19
*BnLAC6-2*	*BnaC04g50890D*	Secretory	C04	48319588:48321669	2081	1743	580	65.01	8.7
*BnLAC7-1*	*BnaA05g29170D*	Secretory	A05	20475705:20479975	4270	1668	555	61.16	9.11
*BnLAC7-2*	*BnaCnng24340D*	Secretory	Cnn	22790373:22794992	4619	1707	568	62.68	9.1
*BnLAC8*	*BnaC02g03650D*	Secretory	C02	1747091: 1749618	2527	1542	513	57.09	8.88
*BnLAC9-1*	*BnaC03g00490D*	Secretory	C03	235782: 239591	3809	1704	567	62.86	6.1
*BnLAC9-2*	*BnaC03g00480D*	Secretory	C03	229247: 232215	2968	1779	592	65.59	6.67
*BnLAC9-3*	*BnaA03g00500D*	Secretory	A03	177682: 180567	2885	1734	577	63.99	6.83
*BnLAC10-1*	*BnaC02g03710D*	Secretory	C02	1776151: 1778498	2347	1680	559	61.18	9.46
*BnLAC10-2*	*BnaAnng13970D*	Secretory	Ann	15018444:15020836	2392	1692	563	61.62	9.46
*BnLAC11-1*	*BnaA10g26680D*	Secretory	A10	16966690:16969252	2562	1686	561	62.54	8.99
*BnLAC11-2*	*BnaC02g03260D*	Secretory	C02	1549295: 1551691	2396	1683	560	62.25	8.49
*BnLAC11-3*	*BnaAnng18410D*	Secretory	Ann	19628923:19631267	2344	1683	560	62.14	8.75
*BnLAC11-4*	*BnaCnng02950D*	Mitochondrion	Cnn	2437726: 2440207	2481	1731	576	64.31	8.88
*BnLAC12-1*	*BnaAnng01310D*	Secretory	Ann	804829: 807062	2233	1698	565	62.58	9.52
*BnLAC12-2*	*BnaA10g25010D*	Secretory	A10	16204889:16207264	2375	1698	565	62.65	9.1
*BnLAC12-3*	*BnaC02g02320D*	Secretory	C02	1036921: 1039163	2242	1695	564	62.50	9.49
*BnLAC12-4*	*BnaC09g49940D*	Secretory	C09	48045040:48047342	2302	1698	565	62.58	9.23
*BnLAC13-1*	*BnaA10g23590D*	Secretory	A10	15550049:15551986	1937	1704	567	63.05	6.65
*BnLAC13-2*	*BnaC09g48310D*	Secretory	C09	47172850:47174803	1953	1701	566	62.86	6.65
*BnLAC14-1*	*BnaC09g47160D*	Secretory	C09	46664083:46666581	2498	1731	576	65.13	9.67
*BnLAC14-2*	*BnaA10g22590D*	Secretory	A10	15188457:15190992	2535	1743	580	65.74	9.74
*BnLAC15-1*	*BnaC02g38340D*	Secretory	C02	41316880:41322634	5754	1692	563	63.60	9.01
*BnLAC15-2*	*BnaAnng08030D*	Secretory	Ann	8068829: 8073341	4512	1680	559	63.42	9.04
*BnLAC15-3*	*BnaA06g30430D*	Secretory	A06	20553666:20556168	2502	1683	560	63.26	9.09
*BnLAC16*	*BnaC09g34170D*	Secretory	C09	37560731:37563386	2655	1713	570	63.07	9.18
*BnLAC17-1*	*BnaA03g09140D*	Secretory	A03	4114497: 4116527	2030	1722	573	63.71	9.28
*BnLAC17-2*	*BnaC03g11450D*	Secretory	C03	5566603: 5568690	2087	1722	573	63.82	9.32
*BnLAC17-3*	*BnaA02g06580D*	Secretory	A02	3141348: 3143631	2283	1719	572	63.47	9.27
*BnLAC17-4*	*BnaA10g12900D*	Secretory	A10	10509237:10513003	3766	1722	573	63.59	9.28

## Supplementary Materials

The following are available online at https://www.mdpi.com/1420-3049/24/10/1985/s1, **Table S1:** Motif sequences of conserved motif analysis, **Table S2:** Putative cis-elements in 45 *BnLACs* promoters, **Table S3:** List of *BnLACs* with putative miRNA target sites, **Table S4:** Expression levels of the *BnLACs* in different tissues and stages of *B. napus*. The values represent the FPKM values, **Table S5:** Expression levels of the *BnLACs* under Cd^2+^, NH_4_^+^ enrichment treatment. The values represent the FPKM values, **Table S6:** Expression levels of 14 selected *BnLACs* in the L(leaf) and S(stem) of the three lines under drought and wound stress, **Table S7:** Expression levels of *BnLAC4, BnCTL2, BnIRX3, BnCESA4, BnIRX1, BnLAC17, BnGAUT12, BnIRX6, BnPGSIP1, BnGLP10* and *BnFLA11* genes in 6 kinds of silique pericarps. The values represent the FPKM values, **Table S8:** Primers used for qRT-PCR analysis.
