# Supplementary material for: Genome-Wide Identification of the LAC Gene Family and Its Expression Analysis Under Stress in Brassica napus
Source: Molecules. 2019 May 23;24(10):1985. doi: 10.3390/molecules24101985 (PMC6571847; doi:10.3390/molecules24101985)
Supplement: Supplementary file 1 [file molecules-24-01985-s001.zip › Supplementary materials/Table S2.docx]

**Table S2. Putative Cis-Elements in *BnLACs* promoters**

| **Laccase** | **Light** | **Hormone responsive** | | | | | | **Stress responsive** | | | |
| --- | --- | --- | --- | --- | --- | --- | --- | --- | --- | --- | --- |
|  |  | **Abscisic acid** | **Auxin** | **Ethylene** | **Gibberellin** | **MeJA** | **Salicylic acid** | **Drought** | **Heat stress** | **Low temperature** | **Wound** |
| BnLAC1-1 | **+** | **+** |  | **+** | **+** |  | **+** | **+** | **+** | **+** |  |
| BnLAC1-2 | **+** | **+** |  | **+** | **+** | **+** | **+** | **+** |  | **+** |  |
| BnLAC2 | **+** |  |  |  | **+** | **+** | **+** |  | **+** |  |  |
| BnLAC3-1 | **+** |  |  |  | **+** |  |  | **+** |  |  |  |
| BnLAC3-2 | **+** |  |  |  | **+** | **+** |  | **+** | **+** |  |  |
| BnLAC3-3 | **+** | **+** | **+** |  | **+** |  | **+** | **+** | **+** |  |  |
| BnLAC3-4 | **+** |  | **+** |  | **+** |  |  |  |  |  |  |
| BnLAC4-1 | **+** |  | **+** | **+** |  |  | **+** | **+** | **+** |  |  |
| BnLAC4-2 | **+** |  |  | **+** |  | **+** | **+** | **+** | **+** |  |  |
| BnLAC4-3 | **+** |  | **+** | **+** |  |  | **+** | **+** | **+** |  |  |
| BnLAC4-4 | **+** |  | **+** | **+** |  | **+** | **+** | **+** | **+** | **+** |  |
| BnLAC5-1 | **+** | **+** |  | **+** |  | **+** |  |  | **+** |  | **+** |
| BnLAC5-2 | **+** |  |  | **+** | **+** | **+** |  | **+** | **+** |  |  |
| BnLAC5-3 | **+** |  |  | **+** | **+** | **+** |  | **+** |  |  |  |
| BnLAC5-4 | **+** |  |  | **+** |  |  |  | **+** | **+** |  |  |
| BnLAC6-1 | **+** |  |  |  |  | **+** |  |  | **+** |  | **+** |
| BnLAC6-2 | **+** |  |  |  |  | **+** |  |  |  |  | **+** |
| BnLAC7-1 | **+** | **+** |  | **+** | **+** |  | **+** | **+** | **+** |  |  |
| BnLAC7-2 | **+** | **+** | **+** |  | **+** |  |  |  | **+** |  |  |
| BnLAC8 | **+** |  |  | **+** |  |  |  |  | **+** |  | **+** |
| BnLAC9-1 | **+** | **+** |  | **+** |  |  |  |  | **+** |  |  |
| BnLAC9-2 | **+** |  | **+** | **+** | **+** |  | **+** |  | **+** |  |  |
| BnLAC9-3 | **+** |  |  | **+** | **+** |  | **+** | **+** | **+** |  |  |
| BnLAC10-1 | **+** |  |  |  | **+** |  | **+** |  |  |  |  |
| BnLAC10-2 | **+** | **+** | **+** |  |  | **+** | **+** |  |  | **+** |  |
| BnLAC11-1 | **+** | **+** |  | **+** | **+** | **+** | **+** | **+** | **+** | **+** |  |
| BnLAC11-2 | **+** | **+** | **+** |  | **+** | **+** |  |  | **+** | **+** |  |
| BnLAC11-3 | **+** | **+** | **+** |  | **+** | **+** |  |  | **+** | **+** |  |
| BnLAC11-4 | **+** |  | **+** |  | **+** | **+** | **+** | **+** | **+** | **+** |  |
| BnLAC12-1 | **+** | **+** |  | **+** |  | **+** | **+** | **+** | **+** |  |  |
| BnLAC12-2 | **+** |  | **+** | **+** |  |  |  | **+** | **+** |  |  |
| BnLAC12-3 | **+** | **+** |  | **+** |  | **+** |  |  | **+** |  |  |
| BnLAC12-4 | **+** |  |  | **+** |  |  |  | **+** | **+** |  |  |
| BnLAC13-1 | **+** | **+** | **+** |  |  | **+** | **+** |  | **+** | **+** |  |
| BnLAC13-2 | **+** | **+** | **+** |  |  | **+** | **+** |  |  | **+** |  |
| BnLAC14-1 | **+** |  |  |  |  | **+** | **+** |  | **+** |  |  |
| BnLAC14-2 | **+** | **+** |  |  | **+** |  |  | **+** | **+** |  |  |
| BnLAC15-1 | **+** |  | **+** | **+** | **+** | **+** |  |  | **+** | **+** |  |
| BnLAC15-2 | **+** | **+** | **+** | **+** | **+** | **+** |  | **+** | **+** | **+** |  |
| BnLAC15-3 | **+** | **+** |  | **+** | **+** | **+** |  |  | **+** | **+** |  |
| BnLAC16 | **+** | **+** | **+** | **+** | **+** | **+** | **+** |  |  | **+** | **+** |
| BnLAC17-1 | **+** |  |  |  | **+** |  |  | **+** |  |  |  |
| BnLAC17-2 | **+** |  |  | **+** |  |  | **+** | **+** | **+** |  | **+** |
| BnLAC17-3 | **+** |  | **+** |  | **+** |  |  | **+** | **+** |  | **+** |
| BnLAC17-4 | **+** |  |  |  | **+** |  | **+** |  |  |  |  |

“+” means contain corresponding promoters
