# Supplementary material for: Genome-Wide Identification of the LAC Gene Family and Its Expression Analysis Under Stress in Brassica napus
Source: Molecules. 2019 May 23;24(10):1985. doi: 10.3390/molecules24101985 (PMC6571847; doi:10.3390/molecules24101985)
Supplement: Supplementary file 1 [file molecules-24-01985-s001.zip › Supplementary materials/Table S3.docx]

**Table S3. List of *BnLACs* with putative miRNA target sites**

| **Gene names** | **Gene ID** | **Predicted miRNA** | **miRNA length** | **Expectation** | **UPE(kcal mol^-1^)** |
| --- | --- | --- | --- | --- | --- |
| BnLAC2 | BnaC04g54790D | miR397a、miR397b | 22 | 1 | 12.49 |
| BnLAC4-1 | BnaA05g06610D | miR397a、miR397b、miR6034 | 21-22 | 1-3 | 12.73-17.24 |
| BnLAC4-2 | BnaC04g07220D | miR397a、miR397b、miR6034 | 21-22 | 1-2 | 11.14-20.15 |
| BnLAC4-3 | BnaA04g21810D | miR397a、miR397b | 22 | 1 | 13.21 |
| BnLAC4-4 | BnaC04g45660D | miR397a、miR397b | 22 | 1 | 13.40 |
| BnLAC11-2 | BnaC02g03260D | miR397a、miR397b | 22 | 1.5 | 15.81 |
| BnLAC11-3 | BnaAnng18410D | miR397a、miR397b | 22 | 1.5 | 15.52 |
| BnLAC16 | BnaC09g34170D | miR397a、miR397b | 22 | 2.5 | 10.85 |
| BnLAC17-1 | BnaA03g09140D | miR397a、miR397b | 22 | 3 | 12.18 |
| BnLAC17-2 | BnaC03g11450D | miR397a、miR397b | 22 | 3 | 12.18 |
| BnLAC17-3 | BnaA02g06580D | miR397a、miR397b | 22 | 0.5 | 20.10 |
